# Usability of Food Size Aids in Mobile Dietary Reporting Apps for Young Adults: Randomized Controlled Trial

**DOI:** 10.2196/14543

**Published:** 2020-04-29

**Authors:** Ying-Chieh Liu, Sheng-Tang Wu, Shan-Ju Lin, Chien-Hung Chen, Yu-Sheng Lin, Hsin-Yun Chen

**Affiliations:** 1 Department of Industrial Design College of Management Chang Gung University Taoyuan Taiwan; 2 Health Promotion Center, Department of Internal Medicine Chang Gung Memorial Hospital Taoyuan Taiwan; 3 Division of Urology Department of Surgery Tri-Service General Hospital and National Defense Medical Center Taipei Taiwan; 4 Cyber Security Technology Institute Institute for Information Industry Taipei Taiwan; 5 Department of Nutrition Therapy Chang Gung Memorial Hospital Taoyuan Taiwan

**Keywords:** portion size measurement, prototype, user-centered design, dietary reporting, mobile health, randomized controlled trial

## Abstract

**Background:**

Young adults are more likely to use self-managed dietary reporting apps. However, there is scant research examining the user experience of different measurement approaches for mobile dietary reporting apps when dealing with a wide variety of food shapes and container sizes.

**Objective:**

Field user experience testing was conducted under actual meal conditions to assess the accuracy, efficiency, and subjective reaction of three food portion measurement methods embedded in a developed mobile app. Key-in–based aid (KBA), commonly used in many current apps, relies on the user’s ability to key in volumes or weights. Photo-based aid (PBA) extends traditional assessment methods, allowing users to scroll, observe, and select a reduced-size image from a set of options. Gesture-based aid (GBA) is a new experimental approach in which the user makes finger movements on the screen to roughly describe food portion boundaries accompanied by a background reference.

**Methods:**

A group of 124 young adults aged 19 to 26 years was recruited for a head-to-head randomized comparison and divided into 3 groups: a KBA (n=42) control group and PBA (n=41) and GBA (n=41) experimental groups. In total, 3 meals (ie, breakfast, lunch, and dinner) were served in a university cafeteria. Participants were provided with 25 dishes and beverages for selection, with a variety of food shapes and containers that reflect everyday life conditions. The accuracy of and time spent on realistic interaction during food portion estimation and the subjective reaction of each aid were recorded and analyzed.

**Results:**

Participants in the KBA group provided the highest accuracy in terms of hash brown weight (*P*=.004) and outperformed PBA or GBA for many soft drinks in cups. PBA had the best results for a cylindrical hot dog (*P*<.001), irregularly shaped pork chop (*P*<.001), and green tea beverage (660 mL; *P*<.001). GBA outperformed PBA for most drinks, and GBA outperformed KBA for some vegetables. The GBA group spent significantly more time assessing food items than the KBA and PBA groups. For each aid, the overall subjective reaction based on the score of the System Usability Scale was not significantly different.

**Conclusions:**

Experimental results show that each aid had some distinguishing advantages. In terms of user acceptance, participants considered all 3 aids to be usable. Furthermore, users’ subjective opinions regarding measurement accuracy contradicted the empirical findings. Future work will consider the use of each aid based on food or container shape and integrate the various advantages of the 3 different aids for better results. Our findings on the use of portion size aids are based on realistic and diverse food items, providing a useful reference for future app improvement of an effective, evidence-based, and acceptable feature.

**Trial Registration:**

International Standard Randomized Controlled Trial Registry ISRCTN36710750; http://www.controlled-trials.com/ISRCTN36710750.

## Introduction

### Background

Young adults are increasingly concerned with healthy eating habits [1-3]. Younger people are more likely to use mobile health (mHealth) apps [4,5], and a broad range of mHealth solutions is available to facilitate self-management of diet, fitness, and weight control [6-9]. Self-managed dietary intake apps are among the most popular apps in the health and fitness category [10]. One major area of mHealth apps has focused on the tracking of daily food intake, nutrient information, and calorie counting based on users’ assessment of their dietary intake [11,12]. However, little research has examined the effectiveness and user acceptance of such apps [13]. A better understanding of how users actually use mHealth apps is required [9].

Assessment accuracy of dietary intake has a significant impact on energy and nutrient intake calculations [14], and many studies have stressed the need to reduce dietary intake measurement errors [15,16]. One of the major challenges inherent in such reporting is the high degree of variation of food shapes, types, and containers in real-world settings [17]. Some technological approaches have sought to facilitate food size measurement, including the use of augmented reality [18,19]; digital photographs [20-22]; comparative food size measurements [23,24]; wearable cameras [25]; and combinations of text, images, and voice recordings in digital devices [26]. However, these studies were mostly based on highly contrived experimental conditions with little or no follow-up in real-world dining contexts. Few studies have compared mobile dietary reporting apps to evaluate the effectiveness of various methods in measuring relative portion size.

### Objectives

This study investigates the effectiveness and limitations of three food size measurement methods in a dietary intake reporting app. Each method has a specific user interaction design and requires different cognitive abilities for task completion. To investigate the practical usage experience, this study seeks to assess the relative effectiveness and acceptability of these different features in tests of young adults reporting their intakes in realistic contexts.

## Methods

### General Overview of the App and its Reporting Procedure

On the basis of user-centered design approaches [27], a new mobile app named DigiDiet (Digital Diet) was developed; it was originally designed for use by patients with metabolic syndrome to improve eating habits. However, DigiDiet functions can also be applied to the dietary tracking and weight management needs of patients with other chronic diseases.

**Figure 1:**
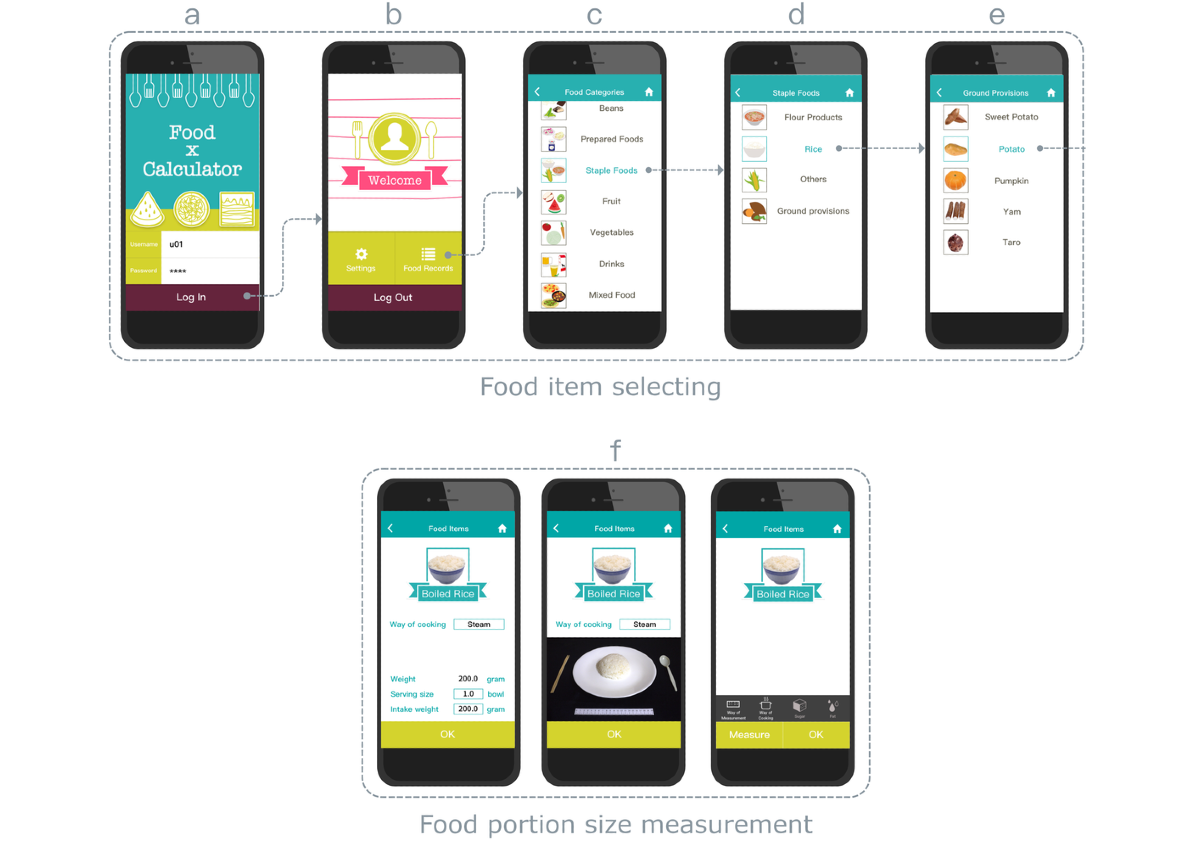
High-level description of dietary intake reporting procedure.

The app’s reporting procedures primarily consist of 2 parts. The first part identifies the food or beverage item to be consumed, using a set of tree-like scrollable lists (steps c and d in Figure 1). The first screen provides a scrollable list of food groups, showing 7 groups at a time. Each field (step c in Figure 1) presents a list headed by a colorful image and text description of the group. The second and third screens (steps d and e in Figure 1), respectively, show subgroups and the main ingredients of the selected item. This part allows users to synthesize a dish using 2 or 3 food items, allowing them to account for a broad range of dishes and food items that may not exist in a predefined tree structure food database (see the study by Liu et al [28] for further details).

The second part (see step f in Figure 1) identifies the portion amount (in terms of portion size or weight) of the item to be consumed. This stage includes 2 distinct functions. First, having selected the main food ingredient, food attributes are displayed in individual tabs. Second, the user taps a particular food attribute (eg, *sugar*, *fat*, and *preparation*) and selects a descriptive value (eg, *half sugar*, *low-fat milk*, and *stir fry*).

Before this research, we had developed prototypes for dietary reporting [28,29]. The major enhancement presented in this study is the inclusion of various portion size aids to help users describe the amount of food consumed. Various users described portion sizes in different ways [11], using weight or volume references, household measures (eg, cup, plate, or bowl), and references against commonly used objects (eg, a credit card) or against hand measures (eg, fist or open palm).

### Three Measurement Aids

Three different measurement designs were developed to support portion measurement. Key-in–based aid (KBA), which is commonly used in many current apps, relies on the user’s ability to key in units of measurement for weight, volume, or household utensils. Photo-based aid (PBA) extends traditional assessment methods [30]. Instead of using life-size paper-based photographs, the aid allows users to scroll, observe, and select a reduced-size image from a set of options. Gesture-based aid (GBA) is a new experimental approach developed based on user finger movements on the screen to roughly describe food volume [31]. Boundaries of the food cross-section and height are prescribed by the user accompanied by a background reference.

**Figure 2:**
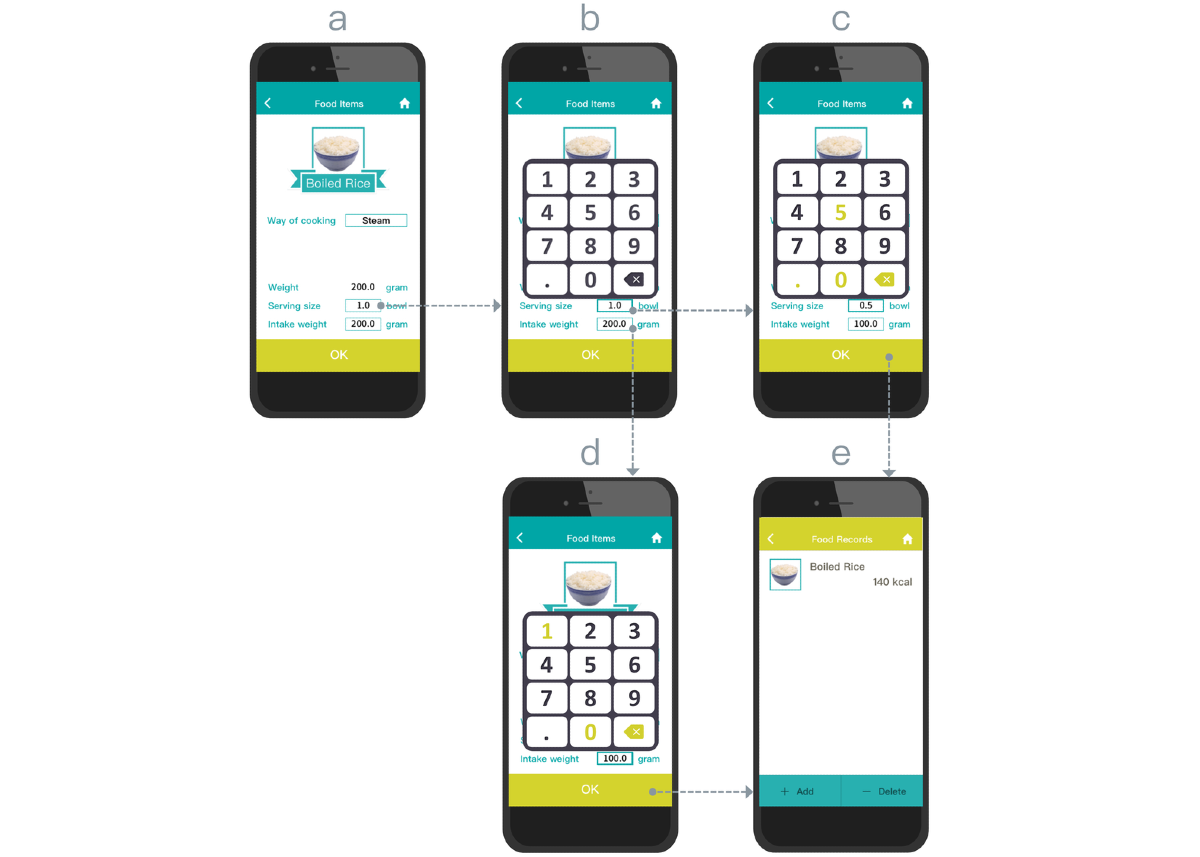
Key-in–based aid interface design and operation.

In the KBA, the user determines the quantity of the food serving (eg, *1* piece, *0.5* cup, or *1.5* slices), and the KBA then outputs a portion weight in grams (see Multimedia Appendix 1). This approach also allows users to enter specific food weights (in grams) to address the converted amount in proportion to the size of the food serving. As shown in step a in Figure 2, the user taps the serving size field to bring up a 12-button field to log the quantity of food size in terms of a specific unit (eg, piece, bowl, or slice). On the basis of the food item and serving size or food weight, the system then outputs the total calories (step d in Figure 2).

**Figure 3:**
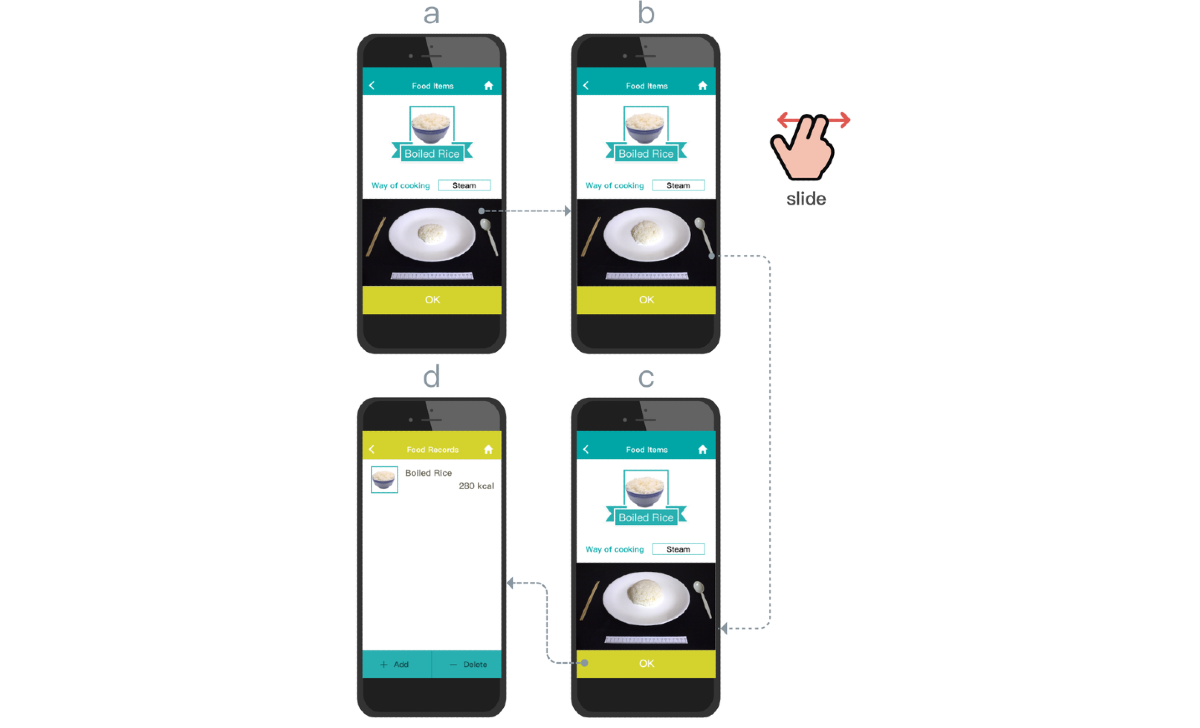
Photo-based aid interface design and operation.

The PBA is an analogical conversion method in which users are provided with life-size food item images on paper [17]. For the purposes of this study, these images are reduced in size and presented on the smartphone screen (see Multimedia Appendix 2). In PBA, users use a one-to-multiple photo selection design to visualize different food quantities. PBA is based on our previous results [29] investigating the potential of applying such measurement methods on mobile devices. We found that this mobile adaptation of PBA can be used to effectively support portion assessment in laboratory testing. This research extends this testing to real-world conditions, including a high degree of variation in food items and container shapes. The pictures of the food items were photographed by the research assistant in accordance with the procedures addressed in the study by Liu et al [29], and these images were integrated into the PBA. Each picture provides a size comparative reference for users. As seen in step c of Figure 3, the user selects one of the several representative photos indicating various proportions. Each photograph includes several objects, including a plate, a pair of chopsticks, a 20-cm ruler, and a spoon (step b in Figure 3). The user then swipes left or right to select the appropriate food portion that best represents the object (steps a-c in Figure 3). Once the user finds an appropriate size relationship, they confirm the selection (step c in Figure 3) and complete the action (step d in Figure 3).

**Figure 4:**
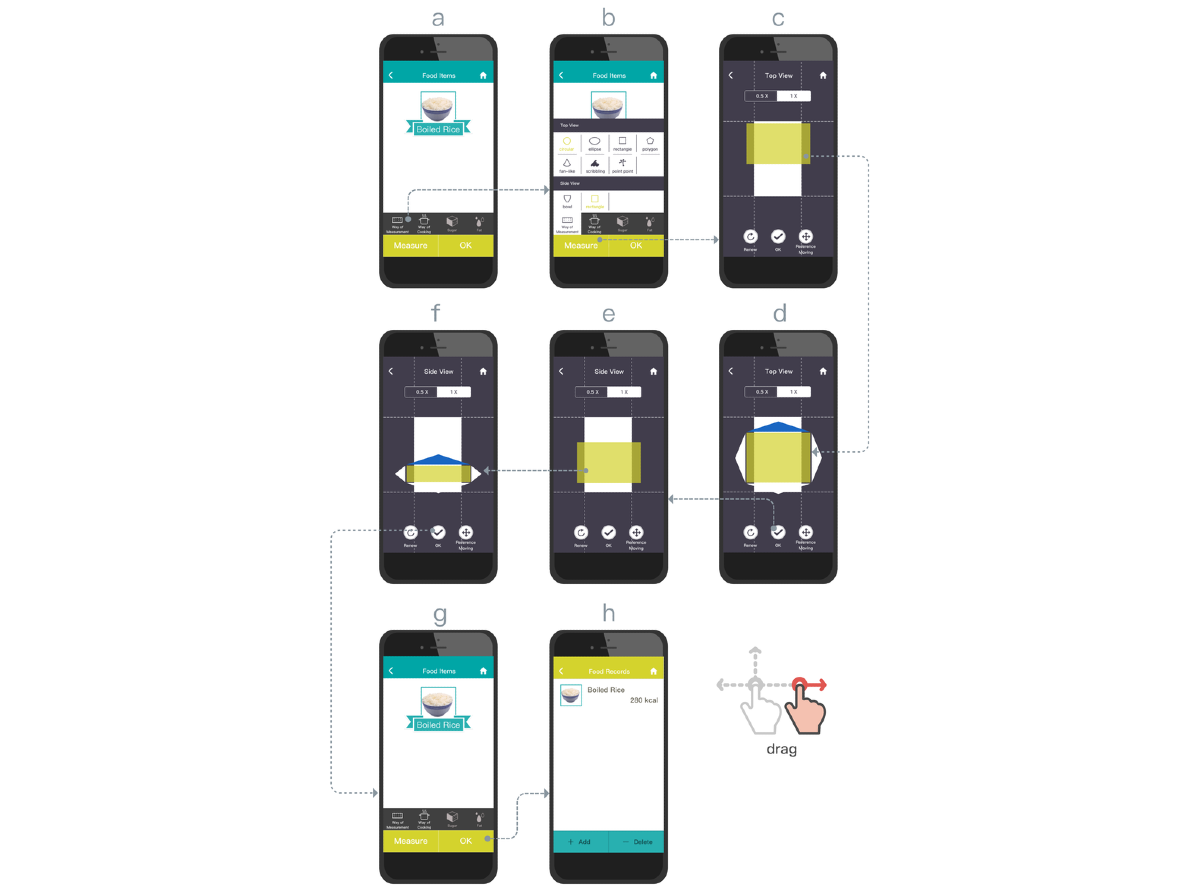
Gesture-based aid interface design and operation.

GBA is a new approach that uses on-screen movements to describe portion volume in 3 dimensions (see Multimedia Appendix 3). GBA is an improvement of our previous research that only described 2-dimensional (2D) food shapes [29]. Portion size is described using a top view and a side view, with the user deforming a basic 2D shape (eg, circle, ellipse, rectangle, polygon, or bowl) to fit the outline from each direction. An irregular 2D shape could be described by repetitive scribbling or dotting to form the desired shape. As shown in step b of Figure 4, the user observes the actual food item and selects an appropriate base shape to initiate measurement, using a credit card as a basis for size comparison. The user then drags and resizes the food item’s top view image to an appropriate ratio of a credit card (steps c and d in Figure 4). Next, the user describes and shades the appropriate area of the side view to address the actual food height, as shown in steps e and f of Figure 4. The estimated volume of the actual food is multiplied by the area and height. The user then submits the query for calculation and storage, as shown in steps g and h of Figure 4.

### Study Design for Evaluation

The study was a single-site, assessor-masked, three-armed, parallel-group randomized controlled trial. Each group used a different measurement function to evaluate and compare their respective performance and user perception. An institutional review board (201601817B0) and the ethics committee at the Chang Gung Memorial Hospital approved the design of this study. The study was implemented in the student cafeteria of the Chang Gung University, Taoyuan, Taiwan, between April 2017 and November 2017.

**Figure 5:**
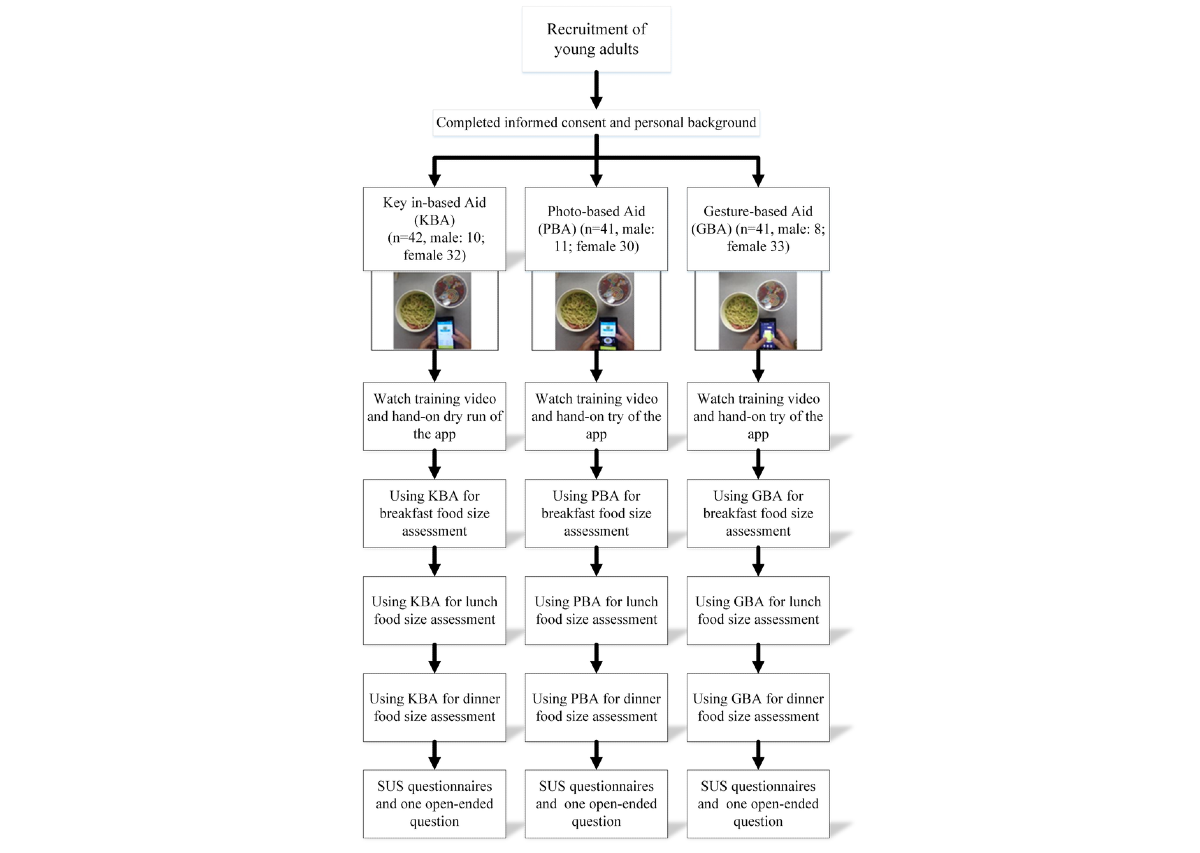
Participant flowchart. SUS: System Usability Scale.

Figure 5 summarizes the experimental process. Respondents were first recruited from among the student population of the Chang Gung University and the Chang Gung University of Science and Technology, both in Taoyuan, Taiwan. Each participant was required to use the developed app for dietary reporting of breakfast, lunch, and dinner in a single day. Eligible participants were university students aged between 19 and 25 years; capable of reading and operating the app on their smartphone; and without diabetes, high cholesterol, or high blood pressure. The exclusion criteria included participants who were currently under any form of dietary control, currently engaged in deliberate weight loss, on medication, or pregnant.

Each participant completed a questionnaire to collect background and baseline data, including age, gender, BMI, academic department, experience with nutrition education, smartphone usage, usage of related apps, and cooking experience (Table 1). Each participant was asked to select intended meal items from a list of 25 food dishes available for consumption in the university cafeteria. Each dish was assessed by a senior dietitian in terms of availability, commonality, preference, and diversity of culinary styles. The food portion, drink containers, and dishes selected were consistent with those commonly seen in the participants’ campus cafeteria (see Multimedia Appendix 4). Considering participants’ eating habits, we deliberately included both Asian and Western-style dishes, specifically Western food for breakfast and dinner and Asian food for lunch. To further reduce the difficulty of using the app, we prepared specific food items that were considered relatively conducive to food size measurement and recording for the first meal (eg, breakfast). Breakfast included a hash brown, ham, a hot dog, and a beverage. For lunch and dinner, the list allowed participants to select a staple food, the main course, 2 types of vegetables, 2 dishes with mixed food ingredients, and 1 beverage.

**Table 1:** Participant characteristics among the key-in–based aid, photo-based aid, and gesture-based aid groups.

Variables		Key-in–based aid (n=42)	Photo-based aid (n=41)	Gesture-based aid (n=41)	Total (N=124)		*P* value
**Gender, n (%)**						.74	
	Male	10 (24)	11 (27)	8 (20)	29 (23.4)		
	Female	32 (76)	30 (73)	33 (81)	95 (76.6)		
**Age (years)^a^**						.19	
	19-20, n (%)	16 (38)	21 (51)	13 (32)	50 (40.3)		
	21-26, n (%)	26 (62)	20 (49)	28 (68)	74 (59.7)		
	Mean (SD)	21.02 (1.54)	20.83 (1.50)	21.34 (1.74)	100		.34
BMI^a^ (kg/m^2^), mean (SD)		21.86 (3.47)	21.86 (2.66)	21.32 (2.90)	100		.65
**Academic department, n (%)**						.54	
	Health care management	19 (45)	21 (51)	21 (51)	61 (49.2)		
	Industrial design	6 (14)	4 (10)	6 (15)	16 (12.9)		
	Nursing	7 (17)	3 (7)	5 (12)	15 (12.1)		
	Industrial business management	3 (7)	1 (3)	4 (10)	8 (6.5)		
	Other	7 (17)	12 (29)	5 (12)	24 (19.4)		
**Experience of using diet** **and nutrition apps** **, n (%)**						.74	
	Yes	10 (24)	8 (20)	7 (17)	25 (20.2)		
	No	32 (76)	33 (80)	34 (83)	99 (79.8)		
**Experience of using Android phones, n (%)**						.91	
	Yes	38 (90)	38 (93)	39 (95)	115 (92.7)		
	No	4 (10)	3 (7)	2 (5)	9 (7.3)		
**Experience of nutrition-related courses** **, n (%)**						.74	
	Yes	20 (48)	21 (51)	23 (56)	64 (51.6)		
	No	22 (52)	20 (49)	18 (44)	60 (48.4)		
**Experience of general health education, n (%)**						.83	
	Yes	26 (62)	28 (68)	27 (66)	81 (65.3)		
	No	16 (38.1)	13 (32)	14 (34)	43 (34.7)		
**Experience in cooking, n (%)**						.87	
	Yes	39 (93)	40 (98)	39 (95)	118 (95.2)		
	No	3 (7)	1 (2)	2 (5)	6 (4.8)		

^a^Age and BMI data were analyzed with analysis of variance.

### Randomization

A 1:1:1 computer randomization was used to equally assign subjects into 1 control group using KBA and 2 experimental groups, respectively, using PBA and GBA to record 3 meals. To ensure that group assignment was random, we used an SAS (SAS Institute Inc) randomization procedure to generate a randomized scheduling program [32]. Sample size determination performance was assessed in terms of accuracy and task completion time (in seconds). The required sample size of each group was determined based on previous similar studies [7]. A minimal sample size of 40 participants was determined for each group.

### Evaluation Outcomes

In total, 3 outcome types were assessed to evaluate the effectiveness of using mobile apps for dietary measurements, including accuracy in terms of the absolute difference between the actual food item weight and system-assessed weight, the respondent’s task duration, and the participant’s perception of efficacy.

#### Accuracy

The first outcome (absolute weight difference) was expressed as a percentage of the difference divided by the actual weight of the food item. The various food items (eg, hash brown, ham, or hot dog for breakfast and chicken leg or pork chop for lunch) were measured before serving. For soft drinks, each cup was filled to a specified level before serving, and the actual weight was determined by the cup size. Ingredients for spaghetti were preweighed by the chef before cooking. Staple foods, vegetables, and dishes with mixed food ingredients were served at a predetermined weight.

#### Task Duration

The assessment duration for each participant was automatically collected through the mobile app. For KBA, the operating duration covers the time from when the participant first begins to input a standard food serving quantity or food weights until the participant taps the *complete* button on the screen. For PBA, the duration covers the time from when the participant tapped the feature button until the participant tapped the *complete* button. For GBA, the duration covers the time from when the participant tapped the measurement button until the participant tapped the *complete* button.

#### Perception

Participants’ perceptions of the utility of each app were measured using the System Usability Scale (SUS) [33], with 10 items scored using a 5-point Likert scale, ranging from 1 (strongly disagree) to 5 (strongly agree). Following the study by Sauro and Lewis [33], a mean SUS score above 64.7 was considered above average. One open-ended question was included to collect each user’s usage experience and suggestions for design improvements.

### Assessment Procedures

The experiment was performed by 2 research assistants. The assessment was scheduled by appointment and implemented on an individual basis. Informed consent was explained to and obtained from each participant. All participants used a 4.7-inch Android smartphone for the test, and all participant trials were conducted on a single day. Each participant first watched an instructional video explaining the operation of the mobile app and the measurement method each participant was assigned to use. Following the written and video instructions, the researchers spent several minutes teaching each participant how to navigate to ensure familiarity with app operation and features and conducted a *dry run*, which involved assessing portion sizes of 4 real food items (bacon, black tea, sweet pepper pork strip, and sausage and spaghetti with cream sauce) and 1 food item (tofu with carrots), which was presented in terms of text and portion description. Participants were encouraged to use the system to assess these items until they felt comfortable with the app operations. For the trial, participants were informed that their time to completion was also a performance consideration. Respondents were asked to record their food items before eating. All participants were asked to record each meal (breakfast, lunch, and dinner) at the time it was served, regardless of whether or how much they actually consumed. Examples of the 3 meals are shown in Multimedia Appendix 4.

### Analysis

We conducted a chi-square test for participant gender and background characteristics and applied analysis of variance for the participants’ age and BMI. Using the difference in weight (measured in grams) and response duration (in seconds) as continuous variables, we applied the Kruskal-Wallis test to the 3 groups and the Mann-Whitney *U* test for multiple two-group comparisons based on the intention-to-treat principle. We also conducted a chi-square test to assess the SUS questionnaire to compare the 3 groups and an independent *t* test for the multiple two-group comparisons. All statistical tests were two tailed, and a *P* value below .05 indicated statistical significance. All statistical analyses were performed using SAS, version 9.1.3 (SAS Institute).

Written responses to the open-ended question were analyzed by 2 research assistants to check the meanings of each participant’s response from different perspectives. Discrepancies between the 2 reviewers were discussed, and a consensus was reached under the project leader’s supervision. Items related to usability and design improvement were highlighted and grouped into specific classes. Individual responses in each category were extracted and counted to obtain a cumulative number for each item.

## Results

### Participant Characteristics

A total of 158 subjects were registered, 135 subjects were scheduled to participate in the experiment, and eventually, 124 subjects completed the study (Figure 3); 11 subjects either failed to record all meals or voluntarily withdrew before completion. The PBA and GBA groups each had 41 respondents, and the KBA group had 42 respondents (see Table 1). Of the respondents who completed the test, 23.4% (29/124) were male; 40.3% (50/124) were aged 19 to 20 years, whereas the remainder were aged 21 to 26 years. The mean BMI of all participants was 21.68 kg/m^2^, with an SD of 3.02. Nearly half (61/124, 49.2%) of the subjects were health care management students, followed by students from miscellaneous departments (24/124, 19.4%), industrial design (16/124, 12.9%), nursing (15/124, 12.1%), and industrial business management (8/124, 6.5%). In terms of previous relevant experience, 95.2% (118/124) of respondents reported having cooking experience, followed by 92.7% (115/124) of respondents who reported using Android operating system phones, 65.3% (81/124) of respondents who reported having general health education, 51.6% (64/124) of respondents who reported taking nutrition-related courses, and 20.2% (25/124) of respondents who reported using diet and nutrition apps. The baseline information distributions did not reveal significant differences among the 3 groups, thereby confirming randomized allocation.

### Weight Comparison Errors

Table 2 summarizes the weight estimation error for all food items among the 3 measurement methods. The results were described in the order of breakfast, lunch, dinner, and beverages, as shown in Table 2.

**Table 2:** Weight comparison error among the key-in based aid, photo-based aid, and gesture-based aid (absolute value).

Meal course and food ingredient			Estimating error in weight (%), mean (SD)			Overall	Key-in vs photo	Photo vs gesture	Key-in vs gesture
			Key-in (n=42)	Photo (n=41)	Gesture (n=41)	*P* value	*P* value	*P* value	*P* value
**Breakfast** (**plate**)									
		Hash browns	18.9 (22.9)	24.6 (17.5)	39.4 (37.1)	.004	.03	.13	.002
		Ham	25.2 (12.9)	33.2 (14.6)	106.6 (315.2)	.05	.001	.84	.09
		Hot dog	53.4 (22.8)	22.9 (8.7)	47.9 (67.9)	<.001	<.001	.008	.003
**Lunch (bento cuboid)**									
	**Staple foods**								
		Rice	39.7 (31.9)	28.0 (14.3)	48.3 (46.6)	.32	.57	.12	.33
		Chow mein	11.9 (19.6)	33.3 (44.2)	67.3 (91.4)	.07	.40	.16	.03
	**Main courses**								
		Chicken leg	37.5 (14.4)	32.9 (12.8)	80.3 (83.9)	.29	.12	.25	.77
		Pork chop	37.4 (9.3)	24.2 (13.8)	78.0 (45.9)	.001	.08	.001	.06
	**Vegetables**								
		Cabbage	79.4 (74.4)	49.5 (53.3)	43.5 (36.8)	.09	.09	.63	.05
		White shoots	77.2 (69.4)	28.0 (6.6)	35.0 (21.4)	.03	.009	.59	.06
		Loofah	49.6 (31.9)	29.2 (20.1)	51.4 (51.0)	.10	.02	.21	.64
		Green beans	55.3 (88.8)	22.9 (8.2)	32.4 (25.6)	.84	.84	.79	.59
		Green pepper	51.8 (50.9)	16.7 (16.0)	44.1 (30.2)	.12	.17	.04	.77
	**Dish with 2 ingredients**								
		Green pepper	238.5 (237.6)	38.5 (18.5)	80.6 (82.5)	.01	.002	.62	.05
		Shredded pork	412.8 (523.3)	7.7 (27.7)	107.0 (124.0)	<.001	<.001	<.001	.26
	**Dish with 2 ingredients**								
		Tomato	152.5 (293.2)	68.5 (48.7)	76.7 (59.5)	.72	.56	.51	.55
		Scrambled eggs	129.4 (105.8)	45.2 (31.7)	90.2 (68.8)	.008	.02	.001	.45
	**Dish with 3 ingredients**								
		Cabbage	106.6 (95.4)	25.9 (19.8)	36.8 (28.5)	<.001	<.001	.13	.008
		Bacon	128.6 (218.0)	3.7 (19.2)	155.7 (159.8)	<.001	<.001	<.001	.07
		Black fungus	332.8 (397.5)	22.2 (42.4)	78.4 (82.7)	<.001	<.001	<.001	.10
	**Dish with 3 ingredients**								
		Fried bean curd	41.9 (40.8)	64.3 (63.3)	210.5 (214.3)	.009	.96	.02	.002
		Green pepper	58.3 (63.4)	26.2 (29.8)	55.0 (62.7)	.18	.13	.11	.64
		Shredded carrot	99.2 (156.3)	31.0 (8.9)	57.3 (30.6)	.03	.13	.003	.60
**Dinner (cylindrical bowl)**									
	**Dish with 2 ingredients**								
		Bacon	60.6 (19.5)	76.5 (8.5)	75.7 (78.0)	.003	<.001	.08	.65
		Spaghetti	41.6 (31.1)	29.6 (25.3)	39.6 (37.7)	.74	.52	.69	.55
	**Dish with 2 ingredients**								
		German sausage	36.1 (31.0)	37.8 (25.9)	192.6 (141.6)	<.001	.65	<.001	<.001
		Spaghetti	59.2 (68.4)	28.5 (25.3)	53.2 (44.8)	.21	.24	.06	.96
**Beverages^a^** **(conical cup)**									
	Orange juice (390 mL)		22.3 (9.7)	38.1 (22.6)	20.3 (10.5)	.04	.06	.03	.32
	Black tea (390 mL)		14.1 (9.6)	51.3 (15.7)	19.4 (10.7)	.001	.003	.002	.29
	Soy milk (390 mL)		31.5 (21.0)	48.7 (15.1)	24.6 (17.9)	<.001	.006	<.001	.07
	Orange juice (490 mL)		9.7 (13.2)	21.0 (14.1)	23.2 (9.1)	<.001	<.001	.19	<.001
	Black tea (490 mL)		10.0 (13.8)	19.3 (10.2)	26.2 (14.2)	.002	.02	.04	.002
	Green tea (500 mL)		19.6 (18.9)	16.0 (9.1)	22.4 (11.7)	.25	.99	.08	.32
	Green tea (660 mL)		7.5 (8.1)	8.9 (21.2)	20.4 (12.2)	<.001	.02	<.001	.009

^a^Beverages include all the beverages for breakfast, lunch, and dinner.

#### Breakfast

Looking at the overall *P* value, all the 3 food items for the breakfast courses were found to incur a significant difference in terms of weight estimation errors. PBA users performed best in the hot dog group (*P*<.001), whereas KBA outperformed PBA or GBA in measuring the hash brown and ham portion sizes.

#### Lunch

All 19 food ingredients present in the food items chosen by the participants were analyzed. As for the staple foods, no significant differences were found. The pork chop in the lunch main course was significantly different (*P*<.001), with PBA outperforming GBA. In the vegetable course, only 1 of the 5 ingredients (ie, white shoots) showed a significant difference (*P*=.03), with PBA outperforming KBA. The other 4 vegetables (ie, cabbage, loofah, green beans, and green pepper) showed no significant differences.

Two lunch items included 2 ingredients each, and analysis of these 4 ingredients found significant differences for 3 (ie, green pepper, shredded pork, and scrambled eggs). Furthermore, another 2 lunch items included 3 ingredients each, and analysis of these 6 ingredients found significant differences for 5 ingredients: cabbage, bacon, black fungus, fried bean curd, and shredded carrot. Of these 5 ingredients, PBA outperformed KBA for cabbage, bacon, and black fungus. PBA also outperformed GBA for all ingredients except cabbage.

#### Dinner

For the dinner food items, each of the 2 spaghetti dishes had 2 ingredients. In total, 4 ingredients were described. The measurement errors for bacon and German sausage showed significant differences (*P*<.01). For bacon, both KBA and GBA outperformed PBA, whereas KBA and PBA outperformed GBA in measuring the German sausage in the two-group comparison.

#### Beverages

Six beverages showed significant differences (*P*<.05), with the exception of the 500-mL beverage (ie, green tea). KBA significantly outperformed the other 2 aids for the two 490-mL beverages (ie, orange juice and black tea). PBA provided the best results for the 660-mL beverages. For the three 390-mL beverages, GBA significantly outperformed PBA, whereas KBA and GBA produced similar results.

### Task Duration

Table 3 summarizes the response time required to estimate the food portion sizes for all food items.

**Table 3:** Portion assessment duration (absolute value).

Meal course and food ingredient			Assessment duration (seconds), mean (SD)						Overall		Key-in vs photo		Photo vs gesture		Key-in vs gesture	
			Key-in (n=42)		Photo (n=41)		Gesture (n=41)		*P* value		*P* value		*P* value		*P* value	
**Breakfast**																
	Hash browns			8.8 (5.8)		8.4 (2.6)		90.5 (39.6)		<.001		.35		<.001		<.001
	Ham			10.9 (7.5)		11.8 (7.5)		83.6 (40.1)		<.001		.33		<.001		<.001
	Hot dog			15.4 (9.7)		10.3 (4.0)		86.4 (41.2)		<.001		.001		<.001		<.001
**Lunch**																
	**Staple food**															
		Rice		9.8 (7.6)		12.1 (6.1)		78.6 (33.3)		<.001		.04		<.001		<.001
		Chow mein		7.2 (3.1)		8.0 (5.8)		86.0 (28.5)		.005		.95		.003		.008
	**Main course**															
		Chicken leg		9.6 (6.4)		9.5 (4.8)		95.5 (34.5)		<.001		.48		<.001		<.001
		Porkchop		9.0 (7.7)		15.3 (4.7)		97.2 (39.1)		<.001		.02		<.001		<.001
	**Vegetables**															
		Cabbage		12.6 (8.1)		11.2 (6.7)		71.9 (29.2)		<.001		.67		<.001		<.001
		White shoots		14.3 (10.0)		8.7 (4.7)		72.6 (32.6)		<.001		.04		<.001		<.001
		Loofah		17.4 (18.1)		9.6 (8.2)		70.1 (33.4)		<.001		.008		<.001		<.001
		Green beans		9.5 (4.6)		9.9 (7.0)		53.2 (16.0)		<.001		.88		.002		<.001
		Green pepper		11.1 (2.9)		11.1 (2.1)		59.7 (30.5)		<.001		>.99		.002		.002
	**Dishes with 2 ingredients**															
		Green pepper+shredded pork		15.8 (8.8)		12.5 (6.3)		70.6 (21.5)		<.001		.31		<.001		<.001
		Tomato+scrambled eggs		22.4 (19.0)		15.0 (7.6)		71.3 (29.6)		<.001		.14		<.001		<.001
	**Dishes with 3 ingredients**															
		Cabbage+bacon+black fungus		23.8 (20.1)		15.3 (7.3)		91.8 (40.3)		<.001		.18		<.001		<.001
		Fried bean curd+green pepper+shredded carrot		33.2 (13.7)		22.6 (12.2)		84.3 (36.4)		<.001		.03		<.001		<.001
**Dinner**																
	Bacon spaghetti		34.1 (15.9)		25.3 (27.1)		97.1 (47.5)		<.001		.002		<.001		<.001	
	German sausage spaghetti		25.5 (11.5)		24.4 (26.7)		91.9 (53.0)		<.001		.09		<.001		<.001	
**Beverages**																
	Orange juice (390 mL)		13.0 (9.6)		15.1 (7.1)		93.6 (29.1)		<.001		.13		<.001		<.001	
	Black tea (390 mL)		13.8 (5.9)		15.5 (5.4)		96.2 (29.1)		<.001		.65		<.001		.001	
	Soy milk (390 mL)		13.9 (9.2)		12.0 (5.6)		94.2 (46.4)		<.001		.99		<.001		<.001	
	Orange juice (490 mL)		11.5 (7.6)		10.3 (3.3)		78.3 (29.4)		<.001		.83		<.001		<.001	
	Black tea (490 mL)		9.3 (5.1)		12.5 (5.6)		84.2 (35.6)		<.001		.05		<.001		<.001	
	Green tea (500 mL)		13.4 (5.2)		12.6 (4.2)		90.2 (42.0)		<.001		.57		<.001		<.001	
	Green tea (660 mL)		13.8 (8.6)		10.6 (3.5)		93.4 (38.7)		<.001		.28		<.001		<.001	

#### Breakfast

All the food dishes (3 for breakfast, 4 for lunch, and 2 for dinner) showed significant differences. In the two-group comparison, KBA and PBA took significantly less time than GBA for all the ingredients, that is, GBA performed worst.

For the breakfast courses, PBA performed best for the hot dog. KBA and PBA performed similarly for the other 2 courses.

#### Lunch

Of the lunch staple food and main courses, KBA performed best for the rice and the pork chop. Of the 5 vegetables, PBA outperformed KBA for the white shoots and loofah. Of the 4 lunch dishes incorporating 2 or 3 ingredients, PBA outperformed KBA for the dish that included fried bean curd, green pepper, and shredded carrot.

#### Dinner

Of the dinner courses, PBA also outperformed KBA for bacon spaghetti.

#### Beverages

All 7 beverage drinks were significantly different. KBA performed best in 1 of the 7 beverages, specifically 490-mL black tea. KBA and PBA performed similarly for the other 6 beverages. GBA performed worst.

### Participants’ Subjective Responses Using System Usability Scale

Table 4 summarizes the response scores for the 3 aids, showing no significant difference. However, KBA significantly outperformed the other 2 aids in terms of usability score (72.8; *P*=.008). In the two-group comparison, KBA significantly outperformed GBA (*P*=.004). In terms of learnability score, no significant difference was found among the 3 aids.

**Table 4:** System Usability Scale score.

Score^a,b,c^	KBA^d^ (n=42), mean (SD)	PBA^e^ (n=41), mean (SD)	GBA^f^ (n=41), mean (SD)	*P* value			
				Overall	KBA vs PBA	PBA vs GBA	KBA vs GBA
Overall score	69.6 (11.6)	67.0 (9.1)	64.0 (12.6)	.08	.25	.23	.04
Usability score	72.8 (11.9)	69.4 (9.5)	64.8 (13.1)	.008	.15	.07	.004
Learnability score	56.5 (21.4)	57.0 (22.7)	61.0 (23.9)	.62	.92	.44	.38

^a^Questionnaires were presented in Chinese.

^b^Mean score for the commercial apps was 64.7.

^c^The questionnaire’s Cronbach alpha of .71 indicated good internal consistency and reliability.

^d^KBA: key-in–based aid.

^e^PBA: photo-based aid.

^f^GBA: gesture-based aid.

### Open-Ended Questions Response

**Figure 6:**
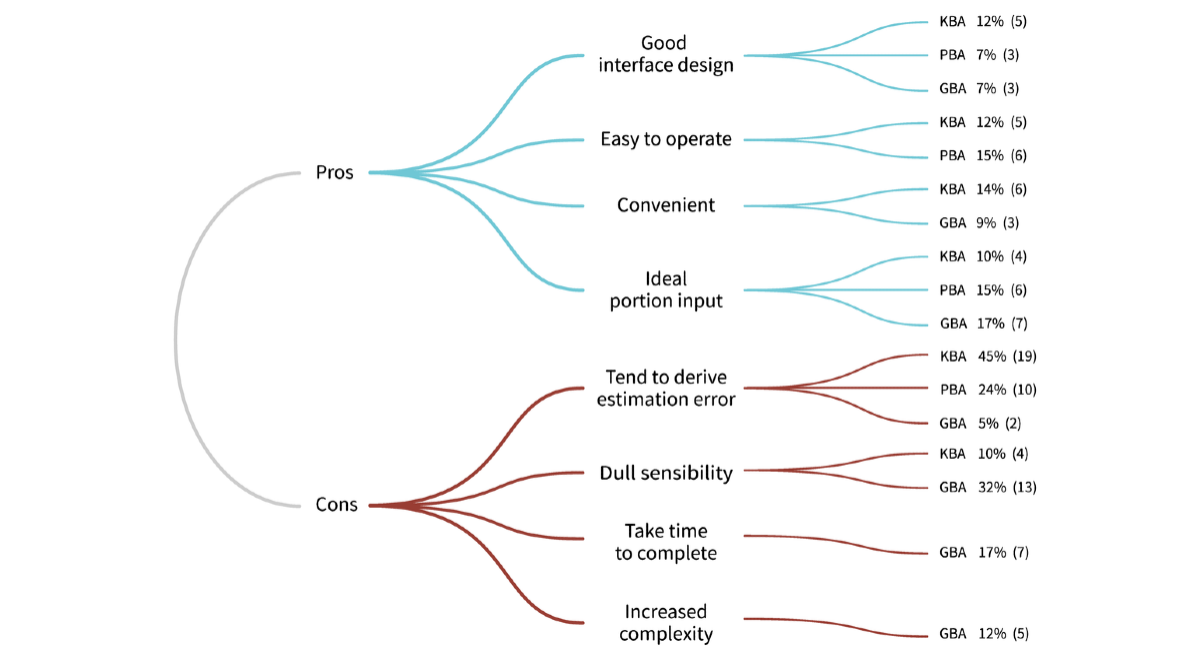
Subjective evaluation. KBA: key-in–based aid; PBA: photo-based aid; GBA: gesture-based aid.

Figure 6 shows the themes identified from the participant evaluations. Participants in all 3 groups cited advantages, including *good interface design* by KBA (5/42, 12%), PBA (3/41, 7%), and GBA (3/41, 7%) and *ideal portion input* by KBA (4/42, 10%), PBA (6/41, 15%), and GBA (7/41, 17%). KBA (5/42, 12%) and PBA (6/41, 15%) were said to be *easy to operate*, whereas KBA (6/42, 14%) and GBA (3/41, 9%) were *convenient*. In terms of drawbacks, KBA (19/42, 45%) participants were most likely to cite *estimation error*, followed by PBA (10/41, 24%) and GBA (2/41, 5%). GBA (7/41, 17%) users cited *takes time to complete*. All 3 aids were cited for being slow to complete the task, whereas GBA (5/41, 12%) alone was cited for *greater complexity*.

## Discussion

### Principal Findings

This research investigated user experiences of the 3 aids by 4 metrics: weight error, operation duration, SUS, and open-ended questions. Using a set group of food and beverage items, this research illustrates the capabilities and limitations of each measurement method for young adults under authentic usage conditions. Our evidence-based findings provide a reference for real-time dietary intake reporting apps. The implications of the findings are discussed, along with the suggestions for further system improvement in the mHealth domain.

### Analysis of Key-in–Based Aid

Similar to the study by Howes et al [12], foods with amorphous shapes showed the highest percent error. Participants in the KBA group produced widely varying results (see Table 2) potentially because KBA relies on prior knowledge when inputting the food serving amount using serving information for input in terms of unit of volume. One potential reason is that authentic food and beverage servings closer to a standard serving size facilitate more accurate guessing and thus result in a higher measurement accuracy. For example, the realistic weight of a chow mein serving is 104 g, which is close to that of a standard bowl (ie, 100 g); thus, participants could simply input *one* in the bowl field to produce an accurate measurement. However, the serving size of the hot dog was around half the standard serving size, thus simply inputting *one hot dog* is likely to result in a high weight error. KBA also produced relatively high degrees of error for dishes with multiple food ingredients (eg, shredded pork, scrambled eggs, bacon, cabbage, and black fungus). That 45% of the group participants considered KBA to *tend to derive estimation errors* reflects this variation.

KBA also tended to outperform the other 2 approaches for standard size beverage containers (eg, 490 mL and 500 mL). However, the participants seemed to be less familiar with the 390-mL container, resulting in a relatively lower accuracy for 390-mL servings of orange juice and soy milk. In addition, the error rate might be affected by the density of the food item using unit of weight for input. For example (see Table 2), soy milk is considerably more dense than water and thus produced a significantly greater error rate (34.9%) than the same volume of black tea (10.3%). A relatively high number of KBA users (see Multimedia Appendix 5) inputted portion weight measurements within a 10% error range for the 490-, 500-, and 660-mL beverages (>53%); hash browns (42%); and chow mein (83%), but this level of accuracy did not extend to all food items. This would address the widely varying results among participants.

### Analysis of Photo-Based Aid

PBA was found to produce relatively higher accuracy rates, although the presentation of the food images differed significantly from the actual food item (Table 2). PBA also performed relatively well for ingredients in the mixed food dishes (eg, shredded pork, bacon, German sausage, cabbage, and black fungus). The research assistant observed that some participants attempted to *dissect* the dish and count the number of chopped vegetables as the basis for choosing an input image. This is an interesting observation worthy of further investigation.

PBA outperformed KBA and GBA for hot dogs potentially because one of its selection items was close to the serving size used in the test. However, selecting the correct representative image does not necessarily result in accurate weight estimation. High weight variety of served food items would raise a substantial challenge for PBA participants. Authentic hash browns, for example, range in weight from 33 g to 64 g, presenting a significant range of error from the 60 g hash brown in the image. This weight variability caused PBA to underperform KBA for both hash browns and ham. The range of authentic foods used in the test would affect the accuracy of PBA. PBA was considerably less able to differentiate between various beverage container sizes possibly because the reference images showed a 660-mL cup filled to different heights, which differed significantly from the different volume beverage containers used in the actual meals. The image for the 660-mL green tea item, however, was identical to the authentic test item and thus elicited a much higher accuracy rate. PBA tended to outperform for those foods that appeared similar to one of the listed images. Similar results were found in our previous research [29]. Training was suggested to further improve PBA accuracy. Additional training in quantification accuracy from digital images would be necessary [12]. In a study by Lee et al [34], images of adolescents eating were first photographed and reported after 14 hours with the support of 2 portion size estimation aids (ie, 2D images [similar to our PBA] and multiple measurement descriptors [similar to our KBA]). Among the tested foods, sausage links were found to have the highest accuracy for both aids. This is similar to our findings for KBA and PBA. However, Lee et al [34] estimated the portion size after 14 hours of food consumption, and the target group (age ranging from 11 to 18 years) differed from that examined here. Gibson et al [23] tested a measurement aid using fist, thumb, or fingertips in comparison with household measures (eg, cups). Using university staff and students as participants, they found that for foods that closely resemble a comparative reference, for example, finger tips would perform better in weight estimation. This is similar to our findings, in that PBA tends to outperform for foods that closely resemble one of the preselected images.

### Analysis of Gesture-Based Aid

GBA requires users to roughly estimate the area and thickness of a food item and then use swiping gestures to describe the approximate food size on the mobile device screen using an accompanying object as a size reference. GBA generally produced relatively inaccurate measurement results. However, the mean estimation error for GBA (Table 2) was relatively consistent for beverages (19.8%-33.2%), vegetables (28.0%-44.7%), and breakfast food items (30.8%-41.5%). GBA also showed a relatively high degree of weight error likely because of the need for the users to determine the length relationship between the food item and the reference object (eg, credit card). GBA errors were also likely because of the need to calculate weight based on food volume, thus requiring users to make 2 independent estimates that compound potential errors. Although GBA suffered from the time spent to complete the measurement, it was considered to be *accurate* based on the subjective opinion. In addition, GBA performed relatively better in some food items when looking at weight accuracy. GBA would be used for some situations when the user was unfamiliar with the food density or the volume size.

### Response Time Comparison of Key-in–Based Aid, Photo-Based Aid, and Gesture-Based Aid

In terms of time efficiency, GBA underperformed compared with KBA and PBA, with respective response times in the ranges of 49 to 95, 7 to 13, and 5 to 16.5 seconds. In addition, no correlation was found between the accuracy rate and the response duration for any of the 3 aids. GBA suffered from longer operational time to estimate the area and height of the food. Apparently, GBA required more time for completion. Similar results were found in our previous research [29], in that GBA-like aid performed significantly worse than PBA in terms of response time.

### Participants’ Perception

Overall SUS scores for KBA and PBA exceeded the average score of 64.80 (Table 4). GBA was marginally close to the average score. Looking at participants’ open-ended responses (see Figure 6), participants in each group considered the used aid to be *ideal portion input*, *easy to operate*, and *convenient*. This was consistent with the overall SUS score. User responses to the open-ended question characterized GBA as *takes time to complete* and *more complex*. This was consistent with the quantitative time efficiency evaluation results in Table 3. Furthermore, GBA was considered to be an *ideal portion input* by 17% of respondents. Only 5% of subjects considered the aid to be *prone to estimation error*, as opposed to 45% for KBA. Participants reported concerns of *no timely response*, *slow to use*, and *more complex* for GBA, indicating a need to further improve its user interface. User perceptions of KBA as tending toward increased estimation error conflicted with the testing result shown in Table 2. However, in terms of accuracy and time duration results, KBA performed relatively better for food items that significantly differed. This apparent contradiction should be investigated in future work. Similarly, König et al [35] also showed that participants’ perceived accuracy did not match their actual accuracy, raising the need for further education in this type of misperception.

### Further Improvements

The design improvement for KBA is a user-friendly design that represents and relates actual food items with the appropriate unit of weight or volume to ease users’ concerns of *estimation error*. Further design improvements in PBA would require providing a selection mechanism to reflect the variations in food shapes and containers. For example, images could be provided to allow users to select various types of containers (eg, cup or bottle), followed by volume (eg, 500 mL or 700 mL) and fullness level (eg, half or one third). GBA is an innovative approach that requires active user input to compare the relative food size and describe food volume. Further design improvements in GBA are needed, such as using multiple finger gestures [31] to improve time efficiency and allow users to select rulers (eg, a 20-mm ruler) rather than relying on a credit card as the point of reference. Future work would also need to resolve technology-related issues that present challenges for food reporting. All three methods inherit certain limitations, for example, visual-based attributes, which do not account for variation in food density. Future improvements would require incorporating nonvisual-based methods or technologies to resolve this limitation.

### Study Limitations

We allowed the KBA group to key in either units of weight (eg, grams) or volume (eg, pieces). These 2 key-in methods could be tested separately in the future to avoid potentially confounding variables. Further tests are required using different target populations (eg, senior citizens or patients with chronic illnesses) whose results may differ from those of young adults. A wider range of authentic foods and longer testing periods could also be included. Another limitation is that the experiment did not consider the possibility that users may not finish certain dishes. To better reflect the realistic eating situation, future research would need to consider the issue of leftovers, for example, to improve the app’s functionality to include the recording of the leftovers.

### Conclusions

Experimental results of young adults using 3 prominent aids showed various strengths and weaknesses. KBA was more accurate for common drink containers or food items that approximate standard serving sizes, whereas PBA performed better for irregular shapes, which closely resemble one of the preselected images, and GBA was better suited for unfamiliar containers or dishes but requires additional design improvement to enhance time efficiency. Thus, to optimize performance, different approaches should be used for different conditions. Participants were queried regarding their subjective impressions of the pros and cons of portion size measurement methods. Concerns were raised regarding estimation errors, device sensitivity, and task complexity. However, participants’ subjective impressions suggest an unfamiliarity with the distinct portion size estimation capability of each aid, and future designs should take this into account. To deal with the complexity of timely collection of dietary data, more work is needed to generate evidence on the appropriateness of each method under specific eating contexts. The user experience results provide scientific evidence for the continued development of related dietary recording apps. Future work could include improvements to design and functionality and the development of new design innovations to enhance effectiveness and convenience. Further studies involving different evaluation metrics are suggested, such as investigating mental loading during dietary recording or considering broader target groups such as low-literacy populations or different eating environments and meal sets.

## Appendix

Multimedia Appendix 1 Mobile app based on key-in–based aid.

Multimedia Appendix 2 Mobile app based on photo-based aid.

Multimedia Appendix 3 Mobile app based on gesture-based aid.

Multimedia Appendix 4 Experimental food samples.

Multimedia Appendix 5 Weight error rates within 10% among the 3 aids.

Multimedia Appendix 6 CONSORT-eHEALTH checklist (V 1.6.1).

## Abbreviations

GBA: gesture-based aid
KBA: key-in–based aid
mHealth: mobile health
PBA: photo-based aid
SUS: System Usability Scale
