# Supplementary material for: Usability of Food Size Aids in Mobile Dietary Reporting Apps for Young Adults: Randomized Controlled Trial
Source: JMIR Mhealth Uhealth. 2020 Apr 29;8(4):e14543. doi: 10.2196/14543 (PMC7221647; doi:10.2196/14543)
Supplement: Multimedia Appendix 4 [file mhealth_v8i4e14543_app4.docx]

**Multimedia Appendix 4. Experimental Food Samples and the Site**

Figure S1~S3 showed the samples of foods in the three meals. All the meals were prepared by the chefs in the campus cafeteria. The weight of each food ingredients were weighed by the research assistants and then prepared for participants. Figure S4 showed the environment of the experiment.


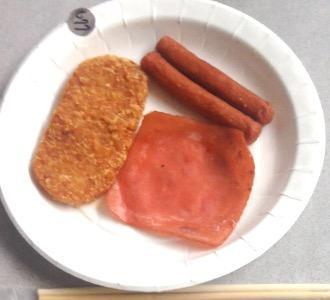


**Figure S1. Three food items and the plate for breakfast experiment**


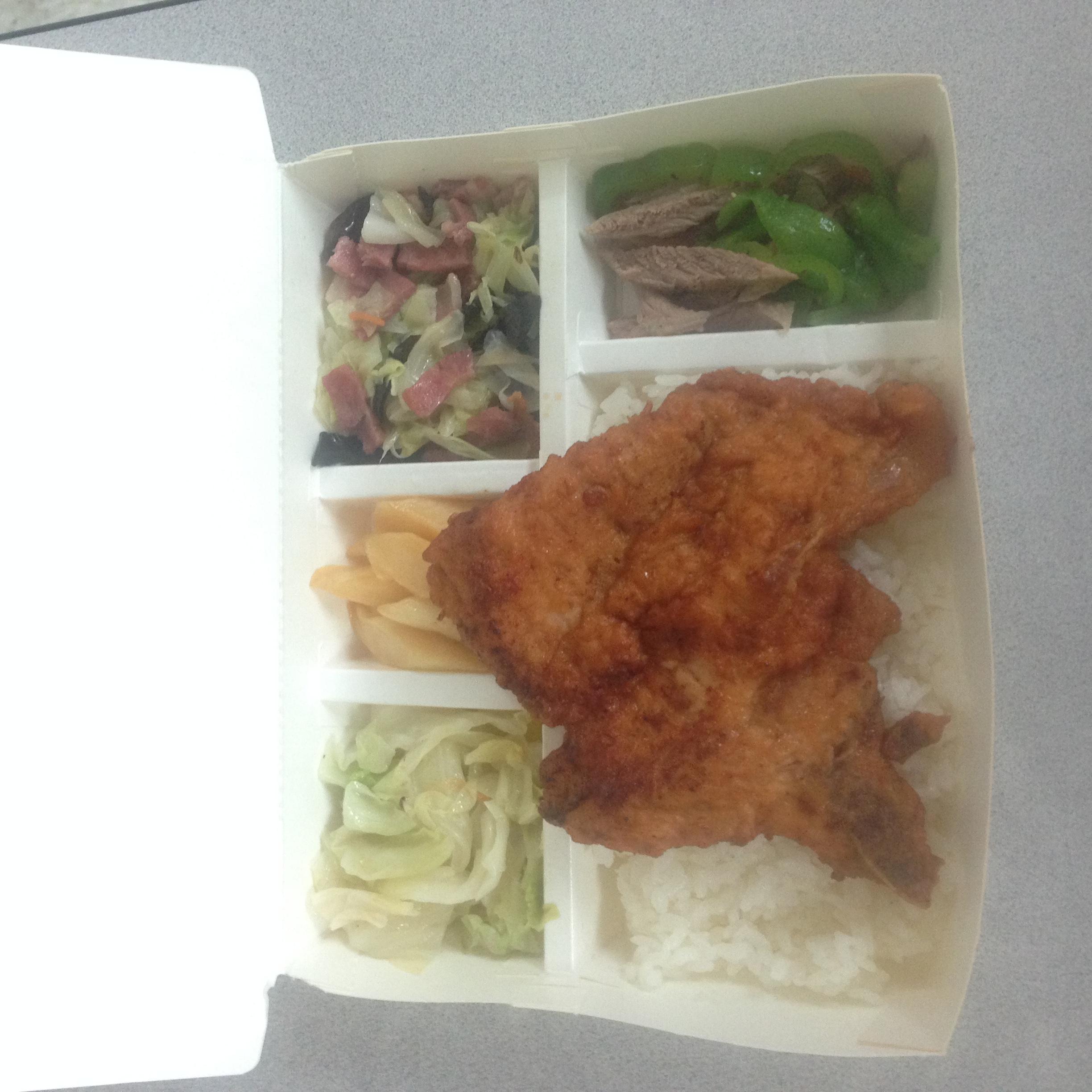


**Figure S2. One staple food, one main course, two vegetables, and two dishes with mixed foods were contained in the bento box for lunch**


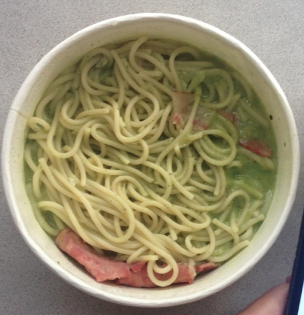


**Figure S3. The main food course of bacon spaghetti contained in a round bowl used for dinner experiment**
