# Supplementary material for: Usability of Food Size Aids in Mobile Dietary Reporting Apps for Young Adults: Randomized Controlled Trial
Source: JMIR Mhealth Uhealth. 2020 Apr 29;8(4):e14543. doi: 10.2196/14543 (PMC7221647; doi:10.2196/14543)
Supplement: Multimedia Appendix 5 [file mhealth_v8i4e14543_app5.docx]

Multimedia Appendix 5 Weight error rates within 10% among the 3 aids

| Meal-course | Food items | KBA Error within 10% range (n) | | PBA Error within 10% range (n) | | GBA Error within 10% range (n) | |
| --- | --- | --- | --- | --- | --- | --- | --- |
| Breakfast | Hash Browns (oral slice) | 42% | (16/38) | 24% | (9/38) | 22% | (7/32) |
|  | Ham (round slice) | 24% | (9/38) | 13% | (5/40) | 13% | (5/38) |
|  | Hot dog (cylindrical capsule) | 0% | (0/41) | 5% | (2/41) | 24% | (8/33) |
| Lunch (bento cuboid) |  |  |  |  |  |  |  |
| -staple foods | Rice | 3% | (1/32) | 20% | (6/30) | 18% | (6/34) |
|  | Chow Mein | 83% | (5/6) | 67% | (6/9) | 20% | (1/5) |
|  |  |  |  |  |  |  |  |
| -main courses | Chicken leg | 9% | (3/33) | 4% | (1/24) | 7% | (2/27) |
|  | Pork chop | 0% | (0/7) | 19% | (3/16) | 0% | (0/11) |
|  |  |  |  |  |  |  |  |
| -vegetables | Cabbage | 6% | (2/32) | 0% | (0/30) | 12% | (3/26) |
|  | White shoots | 7% | (1/15) | 0% | (0/14) | 12% | (2/17) |
|  | Loofah | 0% | (0/18) | 0% | (0/23) | 19% | (4/21) |
|  | Green beans | 27% | (3/11) | 0% | (0/6) | 20% | (2/10) |
|  | Green pepper | 14% | (1/7) | 43% | (3/7) | 13% | (1/8) |
|  |  |  |  |  |  |  |  |
| Lunch |  |  |  |  |  |  |  |
| -Dish with two ingredients | Green pepper+ shredded pork |  |  |  |  |  |  |
|  | Green pepper | 0% | (0/13) | 0% | (0/13) | 8% | (1/13) |
|  | Shredded pork | 8% | (1/13) | 92% | (12/13) | 8% | (1/13) |
| -Dish with two ingredients | Tomato + scrambled eggs |  |  |  |  |  |  |
|  | Tomato | 11% | (3/28) | 0% | (0/28) | 11% | (3/28) |
|  | scrambled eggs | 7% | (2/28) | 0% | (0/28) | 7% | (2/28) |
|  |  |  |  |  |  |  |  |
| Dish with three ingredients | Cabbage + bacon + black fungus |  |  |  |  |  |  |
|  | Cabbage | 10% | (3/29) | 0% | (0/27) | 14% | (4/29) |
|  | Bacon | 17% | (5/29) | 96% | (26/27) | 3% | (1/29) |
|  | Black fungus | 21% | (6/29) | 78% | (21/27) | 3% | (1/29) |
| Dish with three ingredients | Fried bean curd + green pepper + shredded carrot |  |  |  |  |  |  |
|  | Fried bean curd | 50% | (6/12) | 43% | (6/14) | 0% | (0/11) |
|  | Green pepper | 8% | (1/12) | 43% | (6/14) | 27% | (3/11) |
|  | Shredded carrot | 17% | (2/12) | 7% | (1/14) | 0% | (0/11) |
|  |  |  |  |  |  |  |  |
| Dinner (cylinder bowl) |  |  |  |  |  |  |  |
| -Dish with two ingredients | Bacon spaghetti |  |  |  |  |  |  |
|  | Bacon | 0% | (0/21) | 0% | (0/22) | 0% | (0/16) |
|  | Spaghetti | 24% | (5/21) | 45% | (10/22) | 19% | (3/16) |
| -Dish with two ingredients | German sausage spaghetti |  |  |  |  |  |  |
|  | German sausage | 5% | (1/20) | 5% | (1/19) | 0% | (0/17) |
|  | Spaghetti | 10% | (2/20) | 47% | (9/19) | 6% | (1/17) |
|  |  |  |  |  |  |  |  |
| Beverages (conical cup) | Orange juice (390 ml) | 13% | (2/15) | 15% | (2/13) | 20% | (2/10) |
|  | Black tea (390 ml) | 17% | (1/6) | 0% | (0/8) | 18% | (2/11) |
|  | Soy milk (390 ml) | 14% | (3/21) | 0% | (0/20) | 13% | (2/16) |
|  | Orange juice (490 ml) | 76% | (22/29) | 7% | (2/29) | 7% | (2/28) |
|  | Black tea (490 ml) | 74% | (20/27) | 22% | (4/18) | 21% | (4/19) |
|  | Green tea (500 ml) | 53% | (9/17) | 22% | (4/18) | 16% | (3/19) |
|  | Green tea (660 ml) | 64% | (7/11) | 71% | (12/17) | 8% | (1/13) |
